# microRNA-181 promotes prostate cancer cell proliferation by regulating DAX-1 expression

**DOI:** 10.3892/etm.2014.1846

**Published:** 2014-07-16

**Authors:** SHI-JUN TONG, JUN LIU, XIANG WANG, LIAN-XI QU

**Affiliations:** Department of Urologic Surgery, Huashan Hospital Affiliated to Fudan University, Shanghai 200040, P.R. China

**Keywords:** prostate cancer, cell proliferation, microRNA, miR-181, DAX-1

## Abstract

microRNAs (miRNAs) are a class of short noncoding RNA molecules that have a critical role in the initiation and progression of types of human cancer, including prostate cancer. In the present study, the expression of miR-181 in prostate cancer tissues was evaluated and was demonstrated to be significantly upregulated in prostate cancer tissues compared with that in adjacent normal tissues. The results of *in vitro* MTT and BrdU incorporation assays, as well as cell-cycle analysis, indicated that miR-181 overexpression markedly promoted the proliferation of LNCaP cells. Furthermore, miR-181 overexpression was found to promote the progression of LNCaP tumor growth in nude mice. Mechanistic studies demonstrated that dosage-sensitive sex reversal, adrenal hypoplasia critical region, on chromosome X, gene 1 (DAX-1), a negative regulator of androgen receptor in prostate cancer, was inhibited by miR-181 overexpression. Therefore, the results from the present study suggest that miR-181 functions as a growth-suppressive miRNA during prostate cancer development.

## Introduction

Prostate cancer, one of the most common types of cancer in males, has become a major public health concern (1). The molecular pathogenesis of prostate cancer is complicated and remains poorly understood (2). Therefore, the identification of novel molecular mechanisms may help to develop strategies for its diagnosis, treatment and prognosis.

Recent studies have shown that microRNAs (miRNAs) have a critical role in the development of numerous different types of human cancer (3,4). miRNAs regulate multiple genes by targeting mRNAs, resulting in mRNA degradation or translation repression (5). For example, miR-888 is a miRNA secreted by prostate cells that promotes prostate cell growth and migration through the repression of the levels of protein produced by the tumor suppressor genes RBL1 and SMAD4 (6).

Previous studies have demonstrated that the upregulation of hepatic miR-181 promotes the growth, clonogenic survival, migration and invasion of hepatocellular carcinoma cells (7,8). Furthermore, the expression level of miR-181 is significantly associated with overall survival in hematological malignancies and may be an important clinical prognostic factor for patients with hepatocellular carcinoma (9). However, the expression and function of mi-181 in prostate cancer has yet to be elucidated.

Therefore, in the present study, the expression of miR-181 was determined in prostate cancer tissues. In addition, the proliferation of prostate cancer cells overexpressing mi-R181 was analyzed *in vivo* and *in vitro*. Furthermore, the targets of miR-181 were investigated in order to determine the underlying mechanism of miR-181 in prostate cancer.

## Materials and methods

### Tissue samples and cell culture

A total of 20 prostate cancer samples and adjacent normal tissues were obtained from patients who underwent surgery at Huashan Hospital Affiliated to Fudan University (Shanghai, China). The present study was approved by the hospital institutional review board and written informed consent was obtained from each patient. LNCaP cells were provided by the Institute of Biochemistry and Cell Biology of Chinese Academy of Science (Shanghai, China). The cells were cultured in Dulbecco’s modified Eagle’s medium (Invitrogen Life Technologies, Carlsbad, CA, USA) supplemented with 10% fetal bovine serum (Invitrogen Life Technologies), 100 IU/ml penicillin and 100 μg/ml streptomycin sulfate. Cells were incubated at 37°C with 5% CO_2_.

### RNA extraction and quantitative polymerase chain reaction (qPCR)

Total RNA containing miRNA and mRNA was extracted from tissues or cells using TRIzol^®^ reagent (Invitrogen Life Technologies), in accordance with the manufacturer’s instructions. To analyze miR-181 expression, specific stem-loop reverse transcription primers (Invitrogen Life Technologies) were used. In order to determine the transcripts of the interest genes, qPCR was performed using a SYBR Green Premix Ex Taq (Takara, Dalian, China). The primer sequences were listed as follows: DAX-1 sense, 5′-AGCACAAATCAAG CGCAGG-3′, antisense, 5′-GAAGCGCAGCGTCTTC AAC-3′; PSA sense, 5′-CTGCTGCACGTCAGTCAACTA-3′, antisense: 5′-GAGGACTACACTGGTCTGGAAT-3′; CDK1 sense, 5′-AAACTACAGGTCAAGTGGTAGCC-3′, antisense: 5′-TCCTGCATAAGCACATCCTGA-3′ and CDK2 sense: 5′-CCAGGAGTTACTTCTATGCCTGA-3′, antisense: 5′-TTCATCCAGGGGAGGTACAAC-3′.

qPCR was performed by TaqMan MicroRNA assay (Qiagen, Shanghai, China) using the Applied Biosystems 7300 system (Applied Biosystems, Foster City, CA, USA). The PCR conditions included an initial holding period at 95°C for 5 min, followed by a two-step PCR program consisting of 95°C for 5 sec and 60°C for 30 sec for 45 cycles. All samples were normalized against the internal control (U6 small nuclear RNA) and analyzed using the 2^−ΔΔ^Ct method.

### Cell proliferation and cell-cycle assays

The viability of LNCaP cells was determined by assaying the reduction of 3-(4, 5-dimethylthiazol-2-yl)-2, 5-di-phenylte-trazolium bromide (MTT; Beyotime Company, Shanghai, China) to formazan. For the analysis of cell proliferation, cells were seeded onto 24-well plates. For the BrdU incorporation assays, a cell proliferation enzyme-linked immunosorbent assay (ELISA; Beyotime, Shanghai, China) was used to analyze the incorporation of BrdU during DNA synthesis, in accordance with the manufacturer’s instructions. Absorbance was measured at 450 nm using the Spectra Max 190 ELISA reader (Molecular Devices, Sunnyvale, CA, USA). For analysis of the cell cycle, cells were suspended in 0.5 ml solution containing 20 μg/ml propidium iodide and 50 μg/ml RNase, and then analyzed using flow cytometry (Becton Dickinson, San Jose, CA, USA). Histograms were used to represent the percentage of cells in each phase of the cell cycle (G0/G1, S and G2/M).

### microRNA mimics and transfection

Human miR-181 mimics and negative controls (NC) were purchased from Qiagen (Shanghai, China). All transfections of LNCaP cells were performed using Lipofectamine 2000 (Invitrogen Life Technologies, Carlsbad, CA, USA), following the manufacturer’s instructions.

### Western blot analysis

Total cell protein extracts were separated by 10% sodium dodecyl sulfate-polyacrylamide gel electrophoresis, and transferred onto a polyvinylidene difluoride membrane. After blocking with 10% nonfat milk in phosphate-buffered saline, the membranes were immunoblotted with antibodies as indicated, followed by horseradish peroxidase-linked secondary antibodies (Cell Signaling Technology, Inc., Danvers, MA, USA). The signals were detected using a chemiluminescence detection kit (Millipore, Billerica, MA, USA). Anti-dosage-sensitive sex reversal, adrenal hypoplasia critical region, on chromosome X, gene 1 (DAX-1) and anti-GAPDH antibodies were purchased from Abcam (Cambridge, MA, USA). Anti-PSA, CDK1 and CDK2 antibodies were purchased from Santa Cruz Biotechnology Inc. (Santa Cruz Biotechnology, CA, USA). Protein levels were normalized against those of GAPDH (Santa Cruz Biotechnology, Inc.).

### Luciferase reporter assay

cDNA fragments corresponding to the entire 3′-untranslated region (UTR) were amplified by qPCR from the total RNA extracted from LNCaP cells with *Kpn*I and Eco*RI* linkers. The PCR products were cloned downstream of the Renilla luciferase open reading frame of the pMir-Report (Qiagen), which also contained a constitutively expressed firefly luciferase gene that was used to normalize the transfections. For the luciferase reporter assays, the cells were seeded in 24-well plates and harvested 48 h after transfection. The wild-type and mutant 3′-untranslated region fragments from the human DAX-1 gene were cloned into pMir-Report (Qiagen). Mutations were introduced in potential miR-181 binding sites using a site-directed mutagenesis kit (Qiagen). Luciferase values were determined using the Dual-Luciferase Reporter assay system (Promega Corporation, Madison, WI, USA).

### Tumor growth assay

Male BALB/c nude mice, aged 4 weeks, were purchased from the animal center of the Second Military Medical University (Shanghai, China). A total of 2×10^5^ LNCaP cells stably expressing miR-181 or NC were injected subcutaneously into the dorsal flank of the mice. The mice were observed over 5 weeks for tumor formation. The mice were then sacrificed and the tumors were recovered and the wet weight of each tumor was determined. The tumor volume (mm^3^) was calculated according to the following formula: Volume (mm^3^) = 1/2 × length × width^2^. The experimental protocol was approved by the Experimental Animal Care Commission of Huashan Hospital Affiliated to Fudan University.

### Statistical analysis

Differences between groups were analyzed using a Student’s t-test and expressed as the mean ± standard deviation from ≥three independent experiments. P<0.05 was considered to indicate a statistically significant difference. Statistical analyses were performed using GraphPad Prism version .0 software (GraphPad Software, Inc., La Jolla, CA, USA).

## Results

### miR-181 is upregulated in prostate cancer tissues

**Figure 1:**
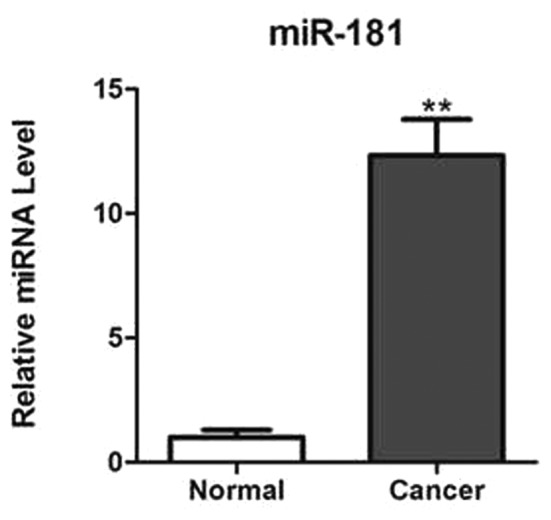
Expression levels of miR-181 in prostate cancer tissues. miR-181 expression was determined by quantitative polymerase chain reaction in human prostate cancer tissues and adjacent normal tissues (n=20). ^**^P<0.01, compared with normal tissues.

The expression of miR-181 was analyzed in prostate cancer tissues and adjacent normal tissues using qPCR. It was found that miR-181 is significantly upregulated in cancer tissues compared with that in normal adjacent tissues, as shown in Fig. 1.

### miR-181 overexpression promotes prostate cancer cell proliferation in vitro

**Figure 2:**
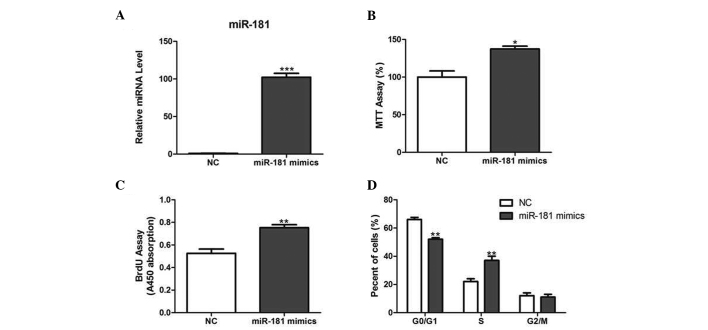
miR-181 overexpression promotes prostate cancer cell proliferation. (A) Expression levels of miR-181 following transfection with miR-181 mimics or NC in LNCaP cells after 24 h. (B) The cell viability of LNCaP cells following transfection with miR-181 mimics or NC in LNCaP cells after 36 h, analyzed using the MTT assay. (C) The proliferative potential of LNCaP cells transfected with miR-181 mimics or NC in LNCaP cells after 36 h, analyzed using the BrdU assay.(D) The cell cycle phases of LNCaP cells transfected with miR-181 mimics or NC were analyzed using flow cytometry. NC, negative controls.

Since miR-181 was found to be upregulated in prostate cancer tissues, the effect of miR-181 on prostate cancer cell growth was investigated. LNCaP cells were transfected with miR-181 mimics or NC (Fig. 2A). The results demonstrated that cell growth was significantly increased in miR-181-overexpressing cells compared with that of their corresponding controls, measured using the MTT and BrdU assays (Fig. 2B and C). Furthermore, miR-181 overexpression decreased the percentage of cells in the G1 phase and increased the percentage of cells in the S phase (Fig. 2D).

### miR-181 overexpression promotes tumor growth in vivo

**Figure 3:**
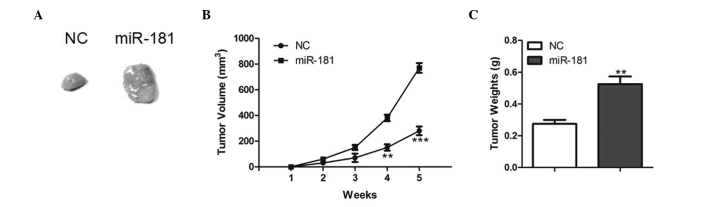
miR-181 overexpression promotes tumor growth *in vivo*. (A–C) LNCaP cells stably transfected with miR-181 or NC were subcutaneously injected into nude mice (n=5 for each group) and tumor growth was monitored. (A) Representative images of the tumors were taken 5 weeks following injection, and the tumor (B) volume and (C) weight were determined. NC, negative control.

To further investigate the function of miR-181 on tumor growth *in vivo*, LNCaP cells with stable overexpression of miR-181 were generated and injected subcutaneously into the dorsal flank of nude mice. Tumor growth was closely monitored for 5 weeks. The tumor size and volume were markedly increased in mice injected with LNCaP cells overexpressing miR-181 compared with those in control mice (Fig. 3A and B). In addition, the average tumor weight was significantly increased by miR-181 overexpression (Fig. 3C), suggesting that miR-181 may promote tumor growth *in vivo*.

### miR-181 targets the DAX-1 3′-untranslated region (3′-UTR) and downregulates its expression

**Figure 4:**
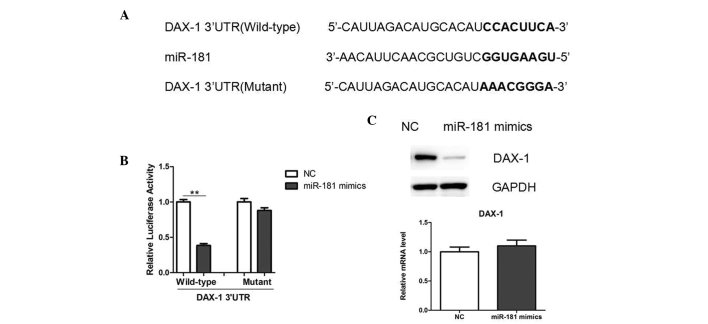
miR-181 represses DAX-1 expression in LNCaP cells. (A) Prediction of miR-181 binding sites in the 3′-UTRs of human DAX-1 gene using TargetScan software. Potential binding sites are highlighted in bold. (B) Luciferase reporter assays in LNCaP cells. Cells were transfected with wild-type or mutant DAX-1 3′-UTR-reporter constructs together with miR-181 mimics or NC. (C) Protein and (D) mRNA levels of DAX-1 were analyzed using western blot analysis and quantitative polymerase chain reaction in LNCaP cells transfected with miR-181 mimics or NC for 36 h. DAX-1, dosage-sensitive sex reversal, adrenal hypoplasia critical region, on chromosome X, gene 1; UTR, untranslated region; NC, negative control.

In order to understand the underlying mechanism, potential targets of miR-181 were determined using TargetScan software. DAX-1 was identified as a potential target of miR-181. Notably, the 3′-UTR of DAX-1 mRNA was observed to contain a complementary site for the seed region of miR-181 (Fig. 4A). To investigate whether DAX-1 may be directly targeted by miR-181, a luciferase reporter vector was constructed, containing the putative miR-181 binding sites within the DAX-1 3′-UTR. The results showed that miR-181 overexpression significantly decreased the luciferase activity, and mutations in the miR-181 binding site from the DAX-1 3′-UTR abolished this effect, suggesting that miR-181 directly inhibited DAX-1 expression by targeting the 3′-UTR (Fig. 4B). Furthermore, miR-181 mimics decreased the endogenous protein levels of DAX-1, as indicated by western blot analysis (Fig. 4C), while the DAX-1 mRNA levels remained unchanged (Fig. 4D). Therefore, these results suggest that miR-181 may negatively regulate DAX-1 expression at the translational level in LNCaP cells.

**Figure 5:**
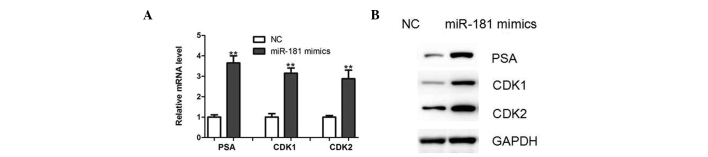
miR-181 regulates down-stream target genes of the androgen receptor. (A) mRNA and (B) protein levels of PSA, CDK1 and CDK2 were analyzed using quantitative polymerase chain reaction and western blot analysis in LNCaP cells transfected with miR-181 mimics or NC for 36 h.PSA, prostate-specific antigen; CDK, cyclin-dependent kinase; NC, negative control.

DAX1 has been previously demonstrated to repress the transcriptional activity of the androgen receptor (AR) in LNCaP cells (10). In the present study, elevated expression levels of AR target genes and proteins, including prostate-specific antigen, cyclin-dependent kinase (CDK) 1 and CDK2, was observed in LNCaP cells overexpressing miR-181 (Fig. 5). In combination, these results further confirm that DAX-1 is an important target gene of miR-181 in prostate cancer cells.

## Discussion

It has been previously demonstrated that several miRNAs are dysregulated in prostate cancer tissues or cell lines, and they have been shown to be associated with prostate cancer progression and disease outcome (11–13). In the present study, it was demonstrated for the first time, to the best of our knowledge, that miR-181 overexpression may promote cell proliferation and cell-cycle progression in LNCaP cells. In addition, miR-181 overexpression was observed to promote the growth of LNCaP tumors in nude mice. Therefore, miR-181 may be an onco-miRNA in the development of prostate cancer.

Furthermore, in the present study DAX-1 was identified as a direct target of miR-181 in prostate cancer cells. DAX-1, a member of the orphan nuclear receptor family, is known to have an important role during development, particularly in gender determination and steroidogenesis (14,15). In humans, DAX-1 gene mutations usually lead to the X-linked congenital adrenal hypoplasia and primary adrenal insufficiency associated with hypogonadotropic hypogonadism (16,17).

With regard to the types of cancer observed in humans, DAX-1 expression has been reported in endocrine and sex steroid-dependent neoplasms, including adrenocortical, pituitary, endometrial and ovarian tumors (18–20). For example, DAX-1 overexpression has been demonstrated to repress estrogen-dependent breast cancer cell proliferation via the inhibition of aromatase expression (19). In addition, DAX-1 expression has been observed to be significantly downregulated in prostate cancer (10). In a previous study, at the molecular level, DAX-1 was demonstrated to interact with the AR and inhibit its nuclear localization. As a result, DAX-1 was found to repress androgen-dependent gene transcription in prostate cancer cells (10). The results from the present study are in accordance with these findings. They demonstrate that miR-181 overexpression causes the upregulation of AR target genes, suggesting that the proliferative role of miR-181, at least in part, may be dependent on androgen signaling.

In conclusion, the present study provides a novel role for miR-181 in prostate cancer cell proliferation. The results suggest that miR-181 may be a potential therapeutic target for the treatment of prostate cancer in the future.
